# Is There a Role for the Non-*Helicobacter pylori* Bacteria in the Risk of Developing Gastric Cancer?

**DOI:** 10.3390/ijms19051353

**Published:** 2018-05-03

**Authors:** Jackie Li, Guillermo I. Perez Perez

**Affiliations:** Department of Medicine, New York University School of Medicine, New York, NY 10016, USA; Jackie.Li@nyumc.org

**Keywords:** *H. pylori*, gastric microbiota, gastric cancer, pro-inflammation

## Abstract

*Helicobacter pylori* is the most abundant bacterium in the gastric epithelium, and its presence has been associated with the risk of developing gastric cancer. As of 15 years ago, no other bacteria were associated with gastric epithelial colonization; but thanks to new methodologies, many other non-*H. pylori* bacteria have been identified. It is possible that non-*H. pylori* may have a significant role in the development of gastric cancer. Here, we discuss the specific role of *H. pylori* as a potential trigger for events that may be conducive to gastric cancer, and consider whether or not the rest of the gastric microbiota represent an additional risk in the development of this disease.

## 1. Introduction

*Helicobacter pylori* was the first bacterium whose presence was associated with increased risk of developing any type of cancer, in this case gastric cancer [1,2]. This was an alarming finding in 1991, because *H. pylori* was, and continues to be, responsible for one of the most prevalent infections in humans globally [3]. However, it was demonstrated that only a minority of the subjects infected with *H. pylori* eventually developed gastric cancer [4]. *H. pylori* infected more than half of the world’s population [3], but it is currently estimated that only between 1% and 3% of those infected individuals develop distal gastric cancer [5,6]. Furthermore, this risk is decreasing gradually with the decrease of *H. pylori* prevalence that has been occurring in the last 100 years [7,8]. This declining risk of developing gastric cancer varies by regions of the world, in which underdeveloped countries possess the larger number of cases for distal gastric cancer. Likewise, these same countries are where the prevalence of *H. pylori* is at its peak [9].

*H. pylori* is responsible for a series of histopathological changes in the gastric mucosa that are recognized as major factors in the development of gastric cancer. This particular series of histopathological changes that lead to gastric cancer are collectively known as Correa’s model [10]. Correa’s model was proposed several years before the discovery and isolation of *H. pylori*, and almost 15 years before the recognition of this organism as a risk factor for gastric cancer [1]. The model was developed exclusively based on histopathological observations related to the gradual progression from a normal gastric epithelium to gastric cancer [11]. Surprisingly, each of the histological changes proposed by Correa in his original paper were later confirmed with *H. pylori* findings, with the progression from a normal gastric mucosa prior to infection followed by chronic superficial gastritis, atrophic gastritis, intestinal metaplasia, dyspepsia and finally gastric carcinoma [12].

Recent progress in the identification of bacteria that colonize different body sites of the human host since birth, collectively named microbiota, has provided detailed insights into the numerous organisms present in the gut and a variety of other body sites, including the oral cavity, skin, lungs, etc. [13]. The human stomach is no exception, and we now know that in addition to *H. pylori*, many other organisms can be present, colonizing the stomach [14]. The relative abundance of bacteria other than *H. pylori* varies depending of the *H. pylori* status of the individual [14]. As a result of these findings, there are now major concerns of the role of bacteria other than *H. pylori* in the development of gastric cancer.

We discuss, in this review, the potential role of the human gastric microbiota in gastric carcinogenesis in the presence or absence of *H. pylori*. We also discuss which *H. pylori* traits contribute to increasing the risk of the development of gastric cancer and whether other members of the gastric microbiota possess similar capabilities.

## 2. Is *H. pylori* a Risk Factor or a True Carcinogen?

The first solid evidence of the association between *H. pylori* infection and gastric cancer was derived from three independent epidemiological studies published in 1991. All three studies reported elevated odds ratios for the development of gastric cancer in subjects who had tested positive for *H. pylori* more than 2 decades before the diagnosis of gastric cancer, when compared with subjects without *H. pylori* infection [15,16,17]. These types of studies have been repeated, and have confirmed the role of *H. pylori* as the most relevant risk factor in the development of gastric cancer [18].

The second type of evidence that implicates *H. pylori* as a risk factor in gastric pathology was obtained from animal models, including piglets, dogs and monkeys. Their normal gastric mucosa was challenged with *H. pylori,* and the development of active and chronic superficial gastritis, and in some cases atrophic gastritis, was confirmed to be associated with *H. pylori* colonization, but not with gastric cancer [19]. The first animal report of *H. pylori* inducing a progression from superficial gastritis to intestinal metaplasia (pre-malignant lesion) was with the use of the Mongolian gerbil model [20]. Some years later, using the same animal model it was confirmed that colonization with *H. pylori* can lead to the development of gastric cancer [21]. More recently, a report summarized the findings of several investigators who had developed a mouse model of gastric cancer in which *H. pylori* challenges were associated with the development of gastric cancer [22]. This model represents a major step forward in the study of *H. pylori* and its role in gastric cancer.

Another approach to documenting the role of *H. pylori* in gastric carcinogenesis is the use of in vitro models. Most of these studies have been dedicated to investigating the role of the major virulence factors of *H. pylori*, including the cytotoxin-associated gene pathogenicity island (CagPAI). It was discovered that *H. pylori* strains expressing the CagPAI are more virulent, and are more frequently isolated from patients with severe clinical outcomes of the infection, including peptic ulcer disease and gastric cancer. The CagPAI is a major chromosomal insertion, encoding around 34 genes, that can be acquired by horizontal transfer [23]. The demonstration that a subset of the CagPAI genes comprises a type IV secretion system was followed by multiple studies confirming the intimate interaction of the transferred CagA protein into the host cells [24]. As a result of these observations, it has been suggested that CagA may be the oncogenic factor in *H. pylori* [25]. It is important to mention that until now, there has not been a single study in vitro that has confirmed the mutagenic ability of *H. pylori* CagA, vacuolating cytotoxin (VacA), or any of its other components. Assessment of mutagenic activity of these factors using methods such as the Ames test is needed to confirm the role of CagA as a true oncogenic protein. The Ames test is a biological assay to assess mutagenic potential of individual compounds using bacteria to test its mutagenic activity. An in vivo experiment in a transgenic mouse model demonstrated that expression of CagA predominantly in the stomach was associated with epithelial hyperplasia, gastric polyps and adenocarcinoma. Systemic expression of CagA was associated with leukocytosis, leukemia and B cell lymphomas [26]. It is important to mention that such neoplastic effects mostly occur after 70 weeks of age, suggesting a chronic process. If *H. pylori* and its components have no direct mutagenic effects in gastric epithelial cells, how does *H. pylori* affect the gastric epithelium in order for it to be considered a major risk factor in gastric cancer development?

To answer this question, we need to revisit the Correa model. Colonization with *H. pylori* occurs early in life, and is maintained for decades or perhaps for the whole life of the colonized individual. The presence of *H. pylori* induces a superficial chronic gastritis influencing the balance between the rate of cellular loss and regeneration [27]. The chronic inflammation induced by *H. pylori* maintains a constant production of a cascade of cytokines, which attracts neutrophils that generate oxidative radicals that have the potential to damage the host DNA. Infection with *H. pylori* has been associated with a reduction in cell replication, and increase in apoptosis, autophagy induction, and endoplasmic reticulum and oxidative/nitrosative stress [28]. These innate immune responses are important to enhance cell survival and proliferation, but as a consequence of the chronic inflammation process due to *H. pylori*, there is a greater chance of acquiring potentially malignant characteristics, which may explain the relevance of this infection as a major risk factor in the development of gastric cancer [28]. This chronic, so-called pro-inflammatory, process appears to be a common denominator and initiator of several chronic diseases [29]. The pro-inflammatory conditions, in concert with the immune response, can lead to a necrotic state. In infections with hepatitis virus B and C, this has been linked to the development of liver cancer. This identical pathophysiological mechanism might be related to *H. pylori* infection and the development of gastric cancer.

Pro-inflammation is a key player in the chronic interaction between *H. pylori* and the host. The original study of El-Omar et al. [30] reported a strong association between pro-inflammatory cytokine polymorphisms and increase risk of developing *H. pylori*-associated gastric cancer. This study provides strong evidence for the influence of host genetic factors and the possibility that the immune necrotic state is relevant for the development of gastric cancer. In addition, these results indicate that an increased inflammatory response, particularly linked with an over-production of pro-inflammatory cytokines and mediators in the Th1 pathway, increase the odds ratio of *H. pylori*-positive individuals developing gastric cancer [31]. Several studies have reported an even greater risk of developing gastric cancer in individuals that are colonized with highly virulent *H. pylori* strains expressing CagA and VacA, and simultaneously carrying cytokine polymorphisms associated with pro-inflammation. Here, the odds ratio for developing gastric cancer increases more than 40-fold [32].

With the improvement of sequencing technology, it has been demonstrated that in addition to *H. pylori*, other bacteria also colonize the gastric mucosa [14]. An important medical question to ask is: What is the role of the non-*H. pylori* microbiota in the process of gastric carcinogenesis? Particularly, what role do these microorganisms play once *H. pylori* has produced gastric atrophy and intestinal metaplasia, and is no longer capable of colonizing the affected gastric mucosa?

## 3. Gastric Microbiota and its Influence on Gastric Carcinogenesis

The recent development of methods for the analysis of 16S rRNA data has provided a clear picture of the bacterial communities present in the human host [33]. Of particular relevance to the detected microbes for this review has been the characterization of the gastric microbiota in patients with and without *H. pylori* infection [34]. One interesting observation was the presence of *H. pylori* sequences in biopsy samples of subjects who had been identified as *H. pylori* negative by most conventional methods [35]. These results show that we now have a powerful and highly sensitive method for detecting bacteria present in very low numbers. However, Kim et al. have established that a key indicator of the biological relevance of detected microbes is the relative abundances of their sequences [35]. In the majority of studies in which subjects were reported negative for *H. pylori* by conventional methods, the sequence data demonstrated the relative abundances of *Helicobacter* to be <2.0%. The clinical relevance of these low-relative-abundance sequences of *H. pylori* remains unsolved. To assess the role of the gastric microbiota as a potential player in *H. pylori*-associated gastric cancer, and to determine the potential interactions between the gastric commensal bacteria and *H. pylori*, it is important to consider the relative abundance of these players, and what type of effect they could produce. In addition, we need to consider the presence of *H. pylori* and its impact on the gastric microbiota, as well as the selectivity that *H. pylori* has in colonizing the gastric epithelium, where any changes affecting the gastric mucosa may affect the ability of *H. pylori* to colonize the stomach.

Another relevant point that needs to be considered is the occurrence of dysbiosis in the gastric community. Most currently available studies report changes in the gastric microbiota in patients with gastric cancer compared to those without [6]. Because of the series of histological changes leading to gastric cancer, the assessment of variations in the gastric microbiota in cancer patients versus non-gastric cancer patients can be clearly predictive. The Correa model describes the histological changes that lead to the progression from a normal gastric mucosa to gastric cancer. The normal acidic pH of the stomach is no longer a main feature of patients with gastric cancer, and they are more likely to present achlorhydria, which is a condition that makes the stomach more permissible for colonization [36,37]. Therefore, the number and type of bacteria colonizing the stomachs of cancer patients will be different from those without cancer.

**Figure 1:**
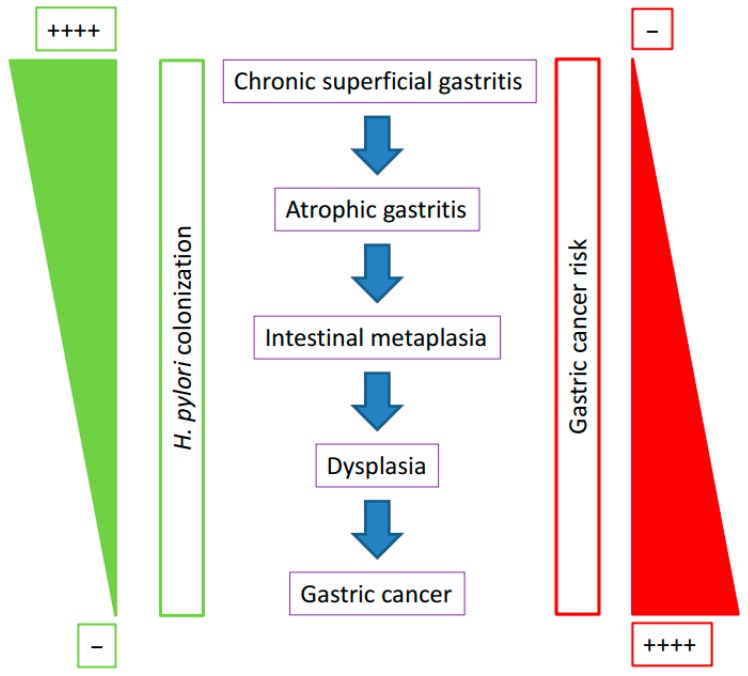
Opposite trends for *H. pylori* density and noncardia gastric cancer risk in relation to the histopathological changes in the human gastric mucosa.

We need to remember that the chronic infection of *H. pylori* in the stomach induces several histopathological changes in the gastric epithelium. In particular, the early pre-malignant changes involving the presence of intestinal metaplasia may precipitate the elimination of *H. pylori* from the human stomach (Figure 1). We believe that those histological changes are critical in the gradual loss of *H. pylori* colonization and the replacement with other microbiota with the capabilities to colonize the modified gastric tissue.

Another important point in the study of the gastric microbiota and its association with *H. pylori* and gastric carcinogenesis is the nature of *H. pylori* colonization, which has a patchy distribution [11]. There are no systematic studies of the gastric microbiota that evaluate whether the presence and composition of the microbiota in the gastric epithelium is homogeneous, or whether it is patchy, like that of *H. pylori*. In order to evaluate this point, multiple gastric biopsies from the same individual need to be studied to determine the true distribution of the gastric microbiota. Furthermore, location of *H. pylori* colonization in the stomach may predict the clinical outcome [38]. It has been suggested that colonization of the antrum region of the stomach is linked with the development of duodenal ulcer. In contrast, the colonization of the body or corpus of the stomach has been associated with a higher risk of developing gastric ulcers and gastric cancer. This topographic distribution of the diseases associated with *H. pylori* may be related to the fact that the enzyme gastric HK-ATPase is found in the gastric parietal cells that are mostly located at the oxyntic gastric glands of the gastric body [39]. The distribution of the acid-producing cells mentioned above might explain why colonization with *H. pylori* of this specific stomach region is a major risk factor for the development of gastric cancer. Future studies aiming to evaluate the role of the gastric microbiota in the development of gastric cancer should specify from which region of the stomach the gastric biopsies are taken.

The relevance of the gastric microbiota in the development of gastric cancer has been confirmed using the transgenic insulin-gastrin mouse model. INS-GAS mice colonized with *H. pylori* alone have a delayed onset of gastric cancer when compared with mice infected with *H. pylori* harboring a gastric microbiota [40]. These results indicate that a gastric microbiota magnifies the effects of *H. pylori* in gastric carcinogenesis but does not necessarily have a direct role in carcinogenesis. *H. pylori* is the major trigger of the histopathological changes leading to gastric cancer and its presence influences the gastric microbiota. In addition, most of the effects related to the development of gastric cancer in *H. pylori* infection are exacerbated if the infected strain is CagA+. However, infection with *H. pylori* CagA+ strains has minor effects on the gastric microbiota, affecting alpha diversity, but not beta diversity or its relative abundance [41].

Most studies of the gastric microbiota have shown that the most dominant phyla are Proteobacteria, Firmicutes, Actinobacteria, Bacteroides, and Fusobacteria [39]. These same phyla are dominant in all the other sites of the human host [33]. This constant prevalence of the same microbes makes it difficult to identify major differences in the microbiota in patients with different clinical outcomes. However, even as some of the studies reported differences in the gastric microbiota between patients with gastritis and patients with gastric cancer [6], it is impossible to determine whether those differences are the cause or the effect of the changes in the gastric epithelium.

Only a few studies have investigated the changes in the gastric microbiota in the pre-malignant stages of gastric cancer (gastric atrophy and intestinal metaplasia) [6,42], or compared the composition of the gastric microbiota in cancer tissue versus non-cancer tissue from the same patient [43]. In the pre-malignant stages, the main finding is that *H. pylori* dominates the microbiota, making it difficult to observe relevant taxa differences between cancer and non-cancer tissue in the same patient.

There are no well-designed studies that implicate the participation of non-*H. pylori* gastric microbiota in the development of gastric cancer. However, in some regions of the world, despite the decline of *H. pylori* infections, increased incidence of gastric cancer has been reported [44,45]. Interestingly, most of these cases have been observed in young adults (<40 years) [46,47,48]. This new epidemiological data may suggest that changes in the gastric microbiota mainly associated with new standards of living, and not infection with *H. pylori*, could be implicated in this age-specific increase in gastric cancer [46]. There are some characteristics of these new cases of non-cardia cancer in young individuals that are different from the traditional non-cardia cancer of all individuals. There is a concordance in most of the reports that young subjects (<40 years) with non-cardia cancer had a more diffuse type than older subjects (>40 years) [46,47,48,49]. Furthermore, contrary to previous statistics in which gastric cancer affected males twice as frequently as females [48], non-cardia cancer in young subjects (<40 years) had an occurrence in women that was higher than or equal to that in men [46,47,48].

A possible explanation for the recent increase in gastric cancer in the USA is perhaps the large immigration of Hispanic populations that has occurred recently [49]. However, the incidence of gastric cancer in Hispanics cannot explain the increased incidence of gastric cancer in young individuals in all regions of the world. Another possibility is that changes in non-cardia cancer related to age and sex might be associated with an increased risk of developing autoimmune gastritis [46]. Autoimmune gastritis was associated with elderly women of Northern European ancestry, but has recently been recognized as a disease in all populations and ethnic groups [50], with a predominance in women and early-age onset, similar to the incidence of gastric cancer in young individuals. This disease could be an alternative explanation for the increased incidence of gastric cancer in young subjects (<40 years). However, autoimmune gastritis occurs in all populations and ethnic groups [51], and does not explain the specific increase of non-cardia cancer exclusively in the white non-Hispanic population in the US [46]. 

A recent Japanese study suggested the assessment of aberrant DNA methylation in gastric tissue as a means to determine risk of gastric cancer [52]. Hypermethylation of DNA sequences in specific genes, such as tumor suppressor genes, has been observed in the gastric mucosa of infected *H. pylori* subjects [28]. Therefore, identification of epigenetic markers for gastric cancer risk might be important for assessing gastric cancer risk. It is imperative to monitor older subjects who are *H. pylori* negative and living in high-prevalence areas of gastric cancer and *H. pylori* infection for potential development of gastric cancer. Two independent studies have recently reported the benefits of antibiotic treatment in reducing the development of gastric cancer particularly in older individuals [53,54]. Both studies found that early treatment had a significant reduction in the risk of developing gastric cancer. In addition, one of the studies found that the risk for gastric cancer gradually increases with increased number of eradication treatments in patients [54].

In conclusion, the published studies support the idea that the presence of *H. pylori* has a major effect on the composition and relative abundance of the gastric microbiota. In the absence of *H. pylori* the gastric microbiota likely contributes to the perpetuation of the inflammatory stimuli. The role of the microbiota as inflammation stimuli is particularly important in patients previously colonized by *H. pylori*. The pre-malignant changes in the gastric epithelium may favor the conditions for bacteria other than *H. pylori* to induce the inflammatory process related to cancer development. The term “point of no return” in the cascade of events that lead to gastric cancer has been associated with patients with intestinal metaplasia and dysplasia, independent of *H. pylori* status, who are at the highest risk of developing gastric cancer [28]. This phenomenon could explain why patients who become spontaneously negative for *H. pylori* continue on the pathway of gastric carcinogenesis.
